# Sono-Enzymatically Embedded Antibacterial Silver-Lignin Nanoparticles on Cork Filter Material for Water Disinfection

**DOI:** 10.3390/ijms231911679

**Published:** 2022-10-02

**Authors:** Lizeth Bermeo, Kristina Ivanova, Leonardo Martín Pérez, Eva Forés, Sílvia Pérez-Rafael, Juan C. Casas-Zapata, Jordi Morató, Tzanko Tzanov

**Affiliations:** 1 UNESCO Chair on Sustainability, ESEIAAT, Universitat Politècnica de Catalunya, 08222 Terrassa, Spain; 2 Grupo de Investigación Ciencia e Ingeniería en Sistemas Ambientales (GCISA), Facultad de Ingeniería Civil, Departamento de Ing. Ambiental, Universidad Del Cauca, Calle 5 No. 4-70, Popayán 190002, Colombia; 3 Grup de Biotecnologia Molecular i Industrial, Departament d’Enginyeria Química, Universitat Politècnica de Catalunya, 08222 Terrassa, Spain; 4 Instituto de Investigaciones en Ingeniería Ambiental, Química y Biotecnología Aplicada (INGEBIO), Facultad de Química e Ingeniería del Rosario, Pontificia Universidad Católica Argentina (UCA), Av. Pellegrini 3314, S2002QEO Rosario, Santa Fe S2002lrk, Argentina; 5 Consejo Nacional de Investigaciones Científicas y Técnicas (CONICET) and Facultad de Ciencias Bioquímicas y Farmacéuticas, Universidad Nacional de Rosario (UNR), Suipacha 531, S2002LRK Rosario, Santa Fe S2002lrk, Argentina

**Keywords:** silver-lignin nanoparticles, laccase, ultrasound coating, cork-based nanocomposite material, antimicrobial activity, water disinfection

## Abstract

Providing clean drinking water is a great challenge worldwide, especially for low-income countries where the access to safe water is limited. During the last decade, new biotechnological approaches have been explored to improve water management. Among them, the use of antimicrobial nanoparticles for designing innovative centralized and decentralized (point-of-use) water treatment systems for microbial decontamination has received considerable attention. Herein, antimicrobial lignin capped silver nanoparticles (AgLNP) were embedded on residual cork pieces using high-intensity ultrasound coupled with laccase-mediated grafting to obtain biofunctionalized nanomaterial. The developed AgLNP-coated cork proved to be highly efficient to drastically reduce the number of viable Gram-negative *Escherichia coli* and Gram-positive *Staphylococcus aureus* in liquid medium. Additionally, the coated-cork was characterized using FTIR-ATR spectroscopy and SEM imaging, and further used as a filter bed in a point-of-use device for water disinfection. The constructed water filtering system significantly reduced the amount of viable *E. coli* and resistant *Bacillus cereus* spores from filtered water operating at increasing residence times of 1, 4, 6, 16, 24, and 48 h. Therefore, the presented results prove that the obtained cork-based antimicrobial nanocomposite material could be used as a filtering medium for the development of water filtration system to control pathogen dissemination.

## 1. Introduction

Microbiologically unsafe water is among the major causes of morbidity and mortality in developing countries. According to the WHO estimations, around 2.1 billion people worldwide still lack access to safe drinking water [1]. Fecal contamination, whether human or animal, is associated with pathogenic microorganisms that pose a serious risk to public health when they reach water supplies or recreational waters. The most frequently found bacteria in water are the enteric bacteria that colonize the human gastrointestinal tract and are eliminated through fecal matter [2]. The presence of *E. coli* provides evidence of fecal contamination and its concentration in wastewater varies from 10^9^ colony forming unit (CFU)/mL to 10^3^ CFU/mL. *E. coli* infection often include severe stomach cramps, diarrhea, and vomiting. Noteworthy, acute diarrheal infections are responsible for the death of approximately 2 million people per year [3], mainly in countries with low sanitation levels. The 6th Sustainable Development Goal aimed to achieve safe and affordable drinking water for the world population by 2030. Therefore, advances in water treatment techniques play a significant role in achieving this goal.

Water disinfection has become one of the safest and low-cost practices for pathogenic organisms’ removal. Chlorine-based disinfectants (e.g., sodium or calcium hypochlorite, chlorine gas, chlorine dioxide) and ozone are among the most common chemicals used for water disinfection [4], providing an effective and robust barrier for pathogens proliferation [5]. However, most of these compounds lead to the formation of toxic byproducts (e.g., trihalomethanes) [5,6,7] which have been related to the development of gastrointestinal, kidney, and bladder disorders, including cancer [8,9,10,11,12,13,14]. In addition, long-term chlorination processes have led to the emergence of chlorine-resistant bacteria [15], especially common pathogenic spores that pose a serious threat to public health [16]. In particular, recent concerns about *B. cereus* poisoning are growing, since this microorganism is a toxin-producing facultative Gram-positive bacterium commonly found in the environment, and thus can contaminate food and water [17,18]. *B. cereus* has the ability to form spores which are highly resistant to external factors (e.g., radiation, disinfectants, heat and other chemicals) [19] due to their structure and chemical composition [20].

Nanomaterials, such as silver nanoparticles (AgNP), titanium dioxide nanoparticles (NPs), and carbon nanotubes have proved to be superior antimicrobials than commonly used disinfectants to kill unwanted microorganisms in water [21,22]. In particular, AgNP are broad-spectrum antibacterial agents whose efficacy depends on their size and morphology. While the mechanism of their antibacterial activity remains unclear, the most widely accepted one involves the perturbation of the bacterial cell wall, resulting in cellular death [23]. Due to their strong bactericidal effect, AgNP have been used to develop point-of-use water purification systems [24,25]. Despite their promising antimicrobial potential even against antibiotic resistant bacteria, AgNP are quite toxic and the release of silver ions (Ag^+^) from silver-containing products to the environment represents an actual global concern [26,27]. In some cases, AgNP also invoke resistance mechanisms in bacteria.

To solve this weakness, new hybrid biopolymer-metal nanoparticulate materials with improved antimicrobial activity and lower toxicity have been designed aiming to achieve AgNP field-scale applications. For example, during AgNP synthesis, the aggressive reducing chemicals have been replaced with biopolymers (e.g., aminocellulose, chitosan, lignin) acting as both reducing and capping agents [28,29,30]. This biopolymer layer controls the release of Ag^+^, reducing toxicity, and inducing a synergistic improvement of the antimicrobial effect [31]. In addition, phenol oxidases such as tyrosinases and laccases, are capable of enhancing the grafting of NPs on different surfaces. These enzymatic approaches have been previously used by our group for the development of lignin-based adhesives for floor coverings, antimicrobial and shrink resistant wool, permanent dyeing of cotton, antifouling coatings on urinary catheters, and biopolymer hydrogels for wound dressing [32,33,34,35].

On the other hand, AgNP functionalization of materials using ultrasound technology improves NPs stability increasing its antibacterial effect. Moreover, ultrasound is a fast and eco-friendly approach for designing durably coated materials [36,37,38]. The versatility of the ultrasound coating has been described in other works imparting antibacterial functionalities to a wide range of surfaces, such as paper, glass, polymers, medical devices, and textiles [38]. In a previous work, we developed a water purification point-of-use device using hybrid Ag-chitosan NPs to effectively control *E. coli* bacterial contamination in water samples [39].

This work extends beyond the described state-of-the art by developing durable and efficient microbicidal nanoenabled coating on cork matrices by depositing hybrid Ag-lignin NPs (AgLNP) in a single-step sono-enzymatic process with laccase. Hybrid AgLNP with broad efficiency against pathogens, including drug resistant representatives [30], were previously prepared by a green synthetic method without the utilization of harsh chemicals or energy-consuming processes and then enzymatically grafted onto cork matrices in a one-pot laccase/high intensity ultrasound process to obtain functional cork material for constructing a point-of-use device for water disinfection. The rationale behind this sono-enzymatic process is in situ laccase-initiated oxidation of the phenolic groups of lignin shell promoting their polymerization and cross-coupling with the nucleophilic groups from the cork matrices. The high reactivity of the quinoid structures obtained upon the phenolics’ oxidation by laccase under ultrasound field will yield durable antibacterial NP-coating on the surface without any pre-treatment.

The enzymatically modified cork nanocomposite material was used as a filter bed in a point-of-use device for the disinfection of water severely contaminated with *E. coli* as an indicator of fecal contamination. Additionally, considering the lack of information on *B. cereus* spore inactivation in drinking water [40,41], we have also determined the capacity of the synthetized AgLNP-coated cork as the filtering material in a point-of-use device to deal with this resistant microbe during water disinfection.

## 2. Results

### 2.1. Characterization of AgLNP

The green synthesis of the AgLNP was carried out in aqueous solution using AgNO_3_ and lignin as both a reducing and capping agent. The phenolic ring and probably the methoxy group from lignin were both involved in the reduction of Ag^+^ to metallic silver (Ag^0^) [42]. The formation of AgLNP after 72 h was confirmed using UV-vis spectrophotometry and TEM imaging (Figures S1 and S2). The particles obtained appeared spherical in shape with a core-shell morphology (i.e., a metallic center and a thin capping layer of lignin). In addition, an average particle size of 36.72 nm and polydispersity of 0.08 nm were determined by dynamic light scattering. The lower polydispersity observed and the negative ζ-potential of −27.07 ± 1.80 mV confirmed the long-term stability of the obtained AgLNP. In a previous work, XPS and FTIR analyses revealed that the mild conditions used at the present work (i.e., low temperature and mild-alkaline pH) during AgLNP synthesis ensure a low extent of lignin oxidation, preserving the phenolic moieties required for the laccase-assisted NPs immobilization onto the cork [43]. All the results herein obtained for AgLNP characterization were in agreement with those previously reported in other works from our group [28,41,43,44].

### 2.2. Sonochemical Coating of Cork with AgLNP

**Figure 1:**
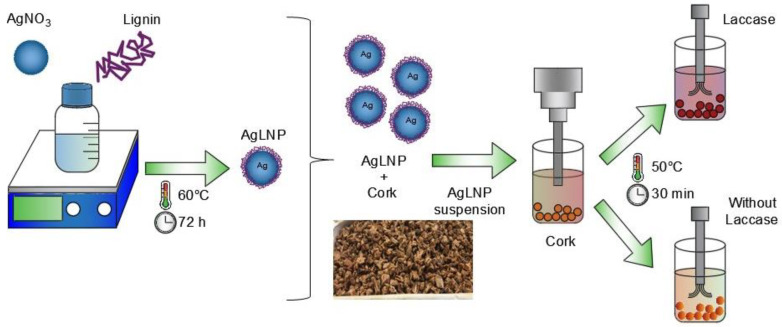
Synthesis and embedding of AgLNP in cork by the sonochemical technique.

Cork granules were sonochemically functionalized in the so called “throwing stones” mode with AgLNP in presence and absence of laccase (Figure 1). In this mode, high intensity ultrasound produces a strong cavitation that favors the homogeneity of the NPs and simultaneously projects the AgLNP towards the surface of the targeted cork material. Therefore, this method is a fast and eco-friendly approach to obtain nanocomposite materials with durable and efficient coatings. On the other hand, the enzymatic immobilization is a very complex process that involves different interactions/reactions between the cork surface and the AgLNP, such as electrostatic interactions, hydrogen bonds, and covalent bonds between laccase-activated cork residues and the AgLNP [44]. Cork chemical composition can vary according to its origin in the range of 40–60% suberin, 10–50% lignin, 5–15% polysaccharides, and 10–25% extractable components (e.g., waxes and tannins). Lignin, suberin, and tannin molecules possess a large number of phenolic groups, which can be enzymatically oxidized into reactive quinones that can further react with amino groups via Michael addition or Schiff base formation [43]. Herein, the phenolic moieties in cork provides bonding sites for covalent immobilization of AgLNP imparting a permanent modification of the material and a more durable antibacterial effect. Our group previously undertook the laccase assisted functionalization of cork with AgNP at standard reaction conditions (e.g., chemical reduction of Ag^+^ to elemental Ag^0^ using NaBH_4_ under vigorous stirring) [43,44]. However, it is a method that requires much more time compared to the ultrasound functionalization used at the present study. Moreover, until this work, to the best of our knowledge, there are no studies demonstrating the feasibility of combining oxidative enzymes with ultrasound coating technology for the immobilization of hybrid metal/phenolic NPs onto phenol-containing materials as cork for the construction of a point-of-use device for water purification.

### 2.3. Characterization of the AgLNP-Coated Cork Nanocomposite Material

**Figure 2:**
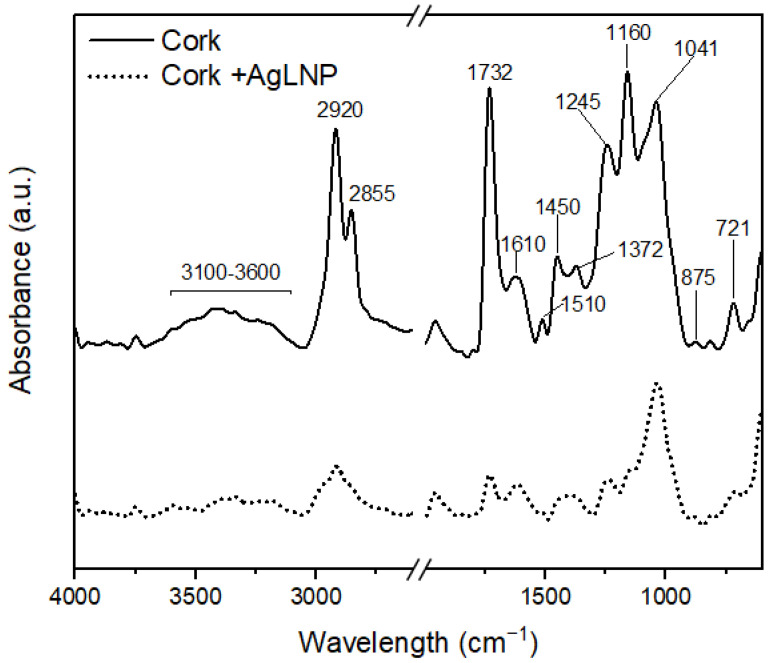
FTIR spectra of unmodified cork (straight lines) and AgLNP-coated cork (dash line).

The FTIR-ATR spectra of unmodified cork and the obtained AgLNP coated-cork material are shown in Figure 2. As can be appraised, clear differences were identified for both spectra. The broad band located at 3100–3600 cm^−1^ corresponding to the stretching vibrations of the –OH group from alcohols and phenols showed a marked intensity reduction in the spectrum of the AgLNP-coated cork, suggesting the oxidation of cork suberin and lignin phenolic groups into quinones [43]. Additionally, the absorption peak at 1041 cm^−1^ corresponding to secondary alcohols was also reduced probably due to the laccase-mediated hydroxyl chain oxidized to α-carboxy [45]. In addition, a visible intensity decrease in the typical suberin fingerprint bands located at 1450, 1245, and 1160 cm^−1^ was observed in the spectrum of AgLNP coated-cork [43]. Regarding this, it was remarkably the reduction of the peak attributed to the C-N stretching of alkyl amines at 1245 cm^−1^. In the other hand, the C-H vibration absorption intensity of aliphatic chains located at 2920 and 2855 cm^−1^ in the spectrum of the uncoated cork showed a significant reduction when AgLNP was added. Moreover, the characteristic bands of ester groups located at 1732 and 721 cm^−1^ showed an intensity reduction with the addition of AgLNP. The peaks corresponding to C-C bond vibration of lignin aromatic rings at 1610 and 1510 cm^−1^ showed an intensity reduction after AgLNP addition. However, the peak located at 875 cm^−1^ appears to be slightly increased, indicating phenolic hydroxyl groups of lignin etherified under the reaction of laccase [45]. Overall, the FTIR-ATR spectra suggest that both suberin and lignin present in the cork nanocomposite material underwent transformations as a consequence of AgLNP interactions and laccase-mediated reactions.

**Figure 3:**
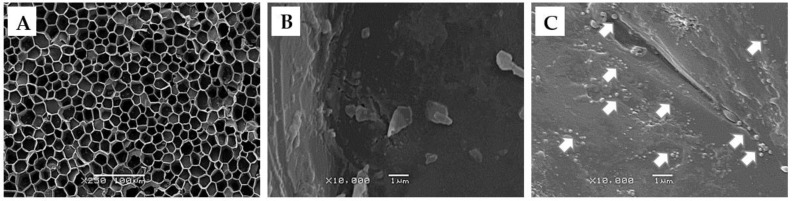
SEM microphotographs showing the ultrastructure of unmodified cork at two magnifications (**A**) = ×250 and (**B**) = ×10,000), and the ultrastructure of the AgLNP coated-cork (**C**). Note the presence of circular nanoparticles attached to the surface of the cork composite material in C (white arrows).

Otherwise, SEM images showed that the AgLNP were configured in agglomerated spherical shapes on the cork surface, thus confirming their successful embedding (Figure 3).

**Figure 4:**
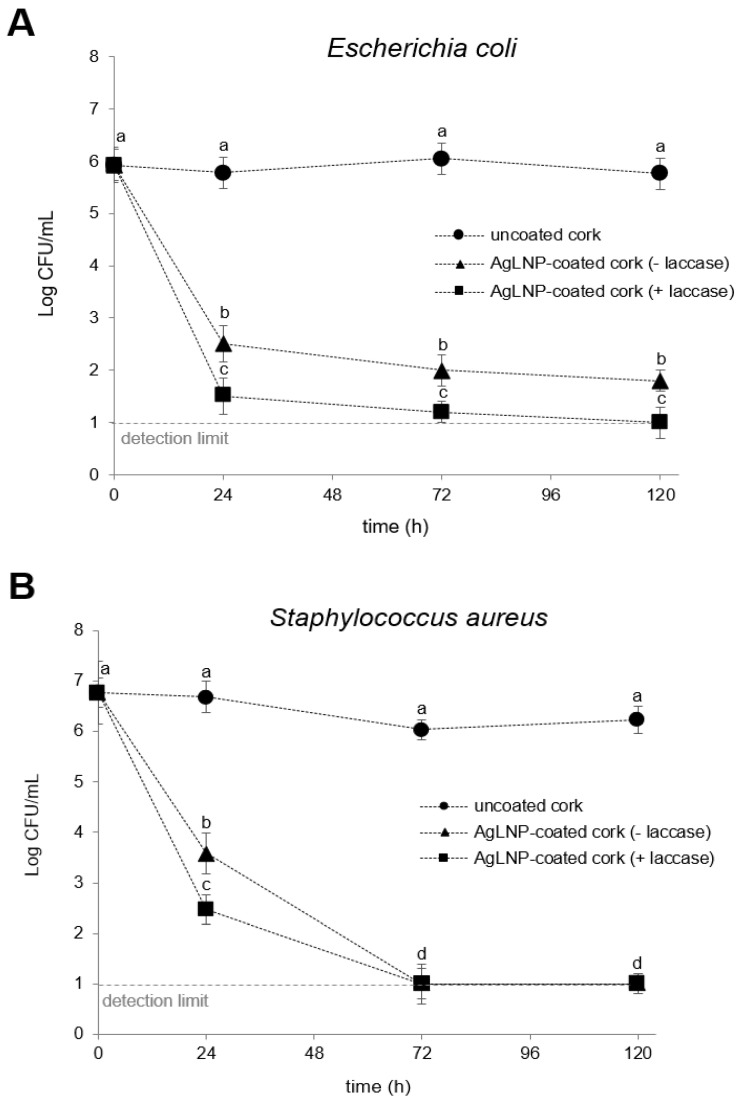
Antibacterial activity in liquid medium of control uncoated cork and AgLNP-coated cork against *E. coli* (**A**) and *S. aureus* (**B**). Different letters represent significant differences (*p* < 0.05) between groups and at increasing exposure time. Data are reported as the mean value ± standard error (S.E.) from three replicates (*n* = 3).

**Figure 5:**
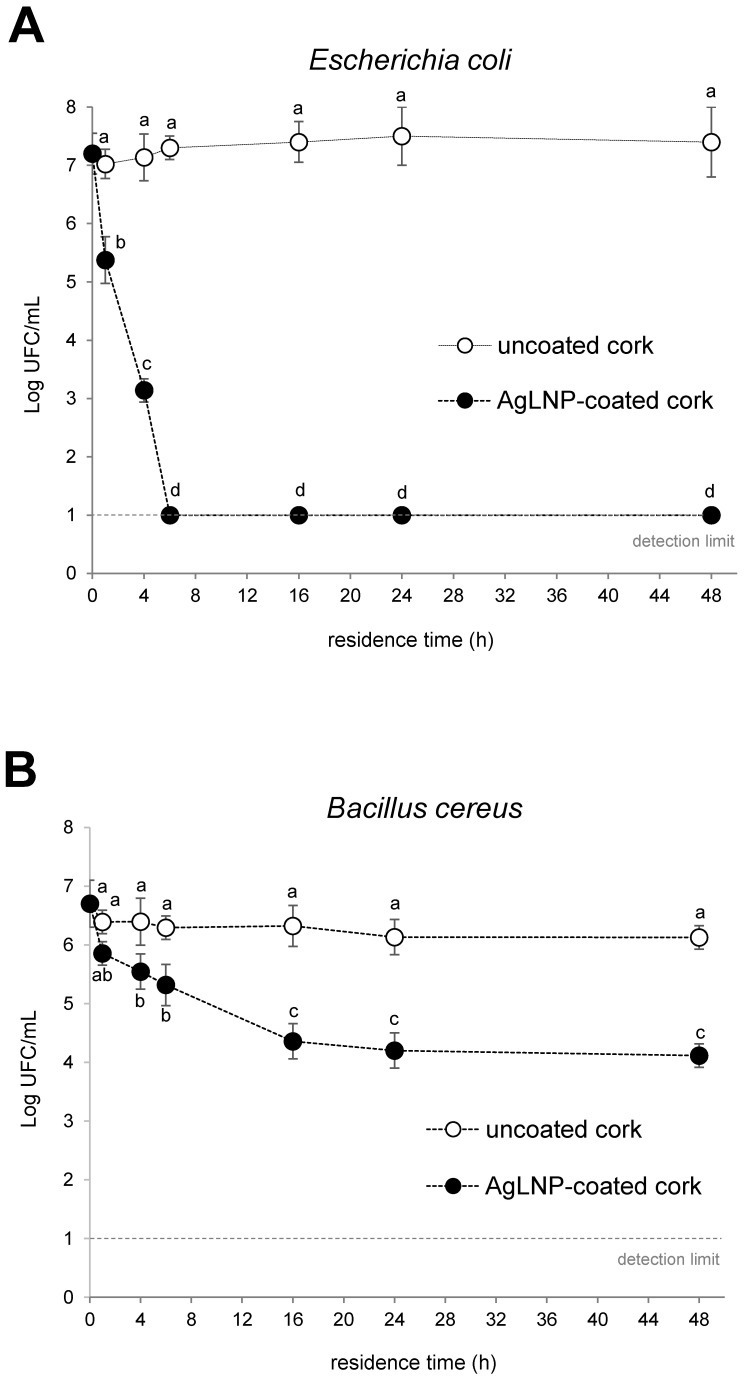
Antibacterial activity against *E. coli* (**A**) and *B. cereus* spores (**B**) of control uncoated- and AgLNP-coated cork acting as the filter medium in a point-of-use device for water disinfection. Different letters represent significant differences (*p* < 0.05) between groups and at increasing residence time; e.g., “a” and “b” are statistically different from each other but not from “ab”. Data are reported as the mean value ± standard error (S.E.) from three biological replicates (*n* = 3) derived from independent experiments.

Finally, Figure 4 shows the antibacterial activity in liquid media of the AgLNP-cork composite material functionalized in the presence and absence of the oxidative enzyme laccase, and tested against Gram-negative *E. coli* (Figure 4A) and Gram-positive *S. aureus*. (Figure 4B). A 4.5-log reduction of *E. coli* counting was obtained for the AgLNP-cork functionalized in the presence of laccase after 24 h treatment. On the other hand, cork nanocomposite material coated without the application of laccase achieved a bacterial reduction of approximately 3.5-log at the same 24 h period (Figure 4A). At the end of the tested period (i.e., 120 h) both obtained materials showed maximal antimicrobial activity. A similar tendency was observed for the Gram-positive *S. aureus*, a 4.5-log reduction was achieved in 24 h for the functionalized AgLNP-cork in the presence of laccase, and a ~3.5-log reduction for the functionalized AgLNP in the absence of the enzyme (Figure 5B). These results may be explained by the fact that laccase is a polyphenol oxidoreductase which can oxidize the phenolic group of lignin into phenoxy radicals through single electron transfer. In this way, the lignin molecules generate a crosslinking structure containing multiple functional groups (e.g., carboxylic, thiol, phenolic and aliphatic hydroxyl groups) that can serve as binding sites for silver ions [45]. Therefore, both a greater amount and greater stability of AgLNP bound to the cork surface could be expected during the coating process in the presence of laccase enzyme. Taking into account these results, it was decided to carry out the sonochemical coating process of the cork in the presence of laccase to further use this nanocomposite material as filter media in a point-of-use device intended for water disinfection.

### 2.4. Determination of Disinfection Efficiency of AgLNP Coated-Cork in a Biofiltration System

The disinfection efficiency of the AgLNP-cork nanocomposite material acting as a biofilter in a point-of-use device for water treatment was evaluated against *E. coli* and *B. cereus* spores (~10^6^–10^7^ CFU/mL initial concentration). As can be appraised in Figure 5A, a 2–4-log reduction in *E. coli* counting from treated water was observed for a residence time of 1 and 4 h, respectively. Notably, a complete bacterial elimination from contaminated water was achieved from 6 h residence time onwards in the systems using the AgLNP-coated cork as the filtering medium (Figure 5A). Several studies indicate that AgNP can adhere to the bacterial cell membrane altering the cellular permeability and microorganism respiratory functions. It is also possible that AgNP not only interact with the surface of the membranes, but also enter the bacteria due to their small size [46].

The phenolic shell of the AgLNP brings multiple benefits not found in the stand-alone AgNP and other metal-based nano-actives including control of the silver ions release, improved bactericidal activity, and biocompatibility. It also provides necessary functional groups for the successful NPs grafting onto material surface. Our group already demonstrated that lignin confers high surface activity to the AgNP and therefore enhanced interaction with the bacterial membrane leading to effective eradication of both Gram-positive and -negative bacteria, including multi-drug resistant clinical isolates, at safe to human cells concentrations. Moreover, AgLNP possess anti-inflammatory properties and, contrarily to conventional antibiotics, do not induce selective pressure for resistance development due to the additional capacity to disrupt the bacterial membrane [28,30]. More noticeably, the AgLNP-coated cork developed in this study was also efficient to reduce the number of viable *B. cereus* spores in the filtering water. These results are very prominent since *B. cereus* spores are highly resistant to extreme conditions. Thus, this microorganism has been associated with serious health issues, including foodborne diseases [47]. As can be seen in Figure 5B, a significant reduction (*p* < 0.05) in *B. cereus* counting was achieved after 4–6 h residence time of contaminated water in the modified cork biofilters. Moreover, the performance of spores’ elimination for the AgLNP-coated cork-based filters increased up to 16 h residence time, reaching a *plateau* that lasted until the end of the tested period (48 h). In addition, a biphasic nature of spores’ elimination from contaminated water can be appraised with a first rapid phase process up to about 12 h, and a slower one from 16 h onwards.

**Figure 6:**
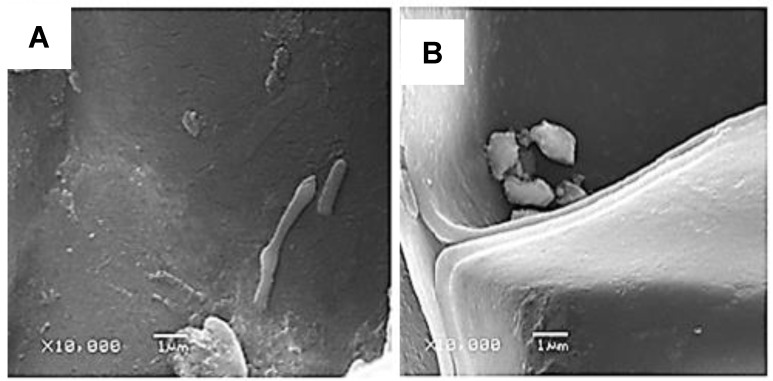
Representative SEM microphotographs of AgLNP-coated cork (**A**) and uncoated cork (**B**) showing microbial attachment to filter medium. Note the characteristic rod-shape of *E. coli* in A and the irregular *B. cereus* spores in B.

Additionally, SEM images of the cork granules used in the present study showed the presence of *E. coli* and *B. cereus* spores adhered to cork surface of both AgNP-coated and unmodified cork (Figure 6). Microbial adsorption to cork surface may occur by a combination of physical and chemical processes (e.g., ionic bonds, chemical chelation, etc.). The obtained FTIR-ATR spectra for coated and uncoated cork presented in Figure 2 show the existence of different functional groups at cork surface, such as carboxyl, amide, and hydroxyl, that could be involved in bacteria biosorption. Moreover, the attachment of spores to cork could be possible since *B. cereus* spores are hydrophobic in nature and can easily adhere to solid surfaces. Additionally, the *B. cereus* spore carries appendages or pili that might be involved in adhesion and may lead to the clustering of spores [39]. Therefore, *B. cereus* spores are not easily removed by cleaning, and they are difficult targets for disinfection. However, the results shown in Figure 5 for the uncoated cork biofilters suggest that bacterial load reduction in the tested water samples due to the potential microbial attachment to cork surface were not significant to be detected by bacteria plate counting. Furthermore, SEM images cannot reveal the cellular state of the attached bacteria, nor the viability of such microorganisms. Therefore, more studies are needed in order to understand the mechanisms involved in bacteria elimination using antimicrobial cork-based nanocomposite materials as filtering media aiming to achieve a rewarding, safe, and more sustainable management of microbial-polluted water.

## 3. Materials and Methods

### 3.1. Materials, Reagents, and Test Bacteria

Granulated cork with mean particle size of ∼0.5 cm was provided by the Catalan Cork Institute (ICSuro, https://www.icsuro.com). Analytical grade silver nitrate (AgNO_3_), lignin (alkali, low sulfonate content with an average Mw ~10.000), hydrochloric acid (HCl), sodium hydroxide (NaOH), and ethanol (EtOH) were purchased from Sigma Aldrich (Madrid, Spain). Laccase (EC 1.10.3.2 *Trametes* sp., Laccase L603P) was provided by Biocatalysts (Nantgarw, UK). Gram-negative *Escherichia coli* (*E. coli* ATCC 25922), and Gram-positive *Staphylococcus aureus* (*S. aureus* ATCC 25923) and *Bacillus cereus* (*B. cereus* ATCC 21769) were used for different antimicrobial assays. For spore formation, 1 mL of an overnight culture of *B. cereus* and 10 mL of sterile phosphate-buffered saline (PBS) (pH 7.0) were mixed and spread (100 µL) onto tryptic soy agar (TSA) plates, which were then incubated for 5 days at 37 °C. After incubation, spores were collected by scraping the agar surface with a sterile swab, suspended in sterile distilled water, and washed three times by centrifugation (10,000 rpm, 5 min). Then, the pellet was resuspended in 10 mL of EtOH and treated with UV-light for 10 min to eliminate vegetative non-sporulated bacteria. Finally, spores were washed again by centrifugation (10,000 rpm, 5 min) and the pellet was resuspended in 1 mL of sterile PBS (pH 7.0) to ~10^7^ spores/mL. TSA, Baird-Parker selective media, TBX (Tryptone Bile X-Glucuronide) chromogenic agar media, and other reagents for cell culture studies were purchased from Sigma-Aldrich (Madrid, Spain).

### 3.2. Preparation and Characterization of AgLNP

Lignin suspension (1% *w*/*v*) was prepared in distilled water and adjusted to pH 5.5 with 5 M HCl using a calibrated electrode. Then, 20 mL of 2 mg/mL AgNO_3_ aqueous solution were added to 30 mL of 1% (*w*/*v*) lignin suspension. The reaction was allowed to proceed in the absence of light at 60 °C for 72 h under magnetic stirring. UV-vis spectrum of the final suspension was collected in the range 200–600 nm using a microplate reader Infinite M200 (Tecan, Austria) to monitor AgLNP formation (Figure S1). The size and morphology of the AgLNP were analyzed by high-resolution transmission electron microscope equipped with energy dispersive X-ray spectroscopy (JEOL JEM-2100 LaB6 TEM). Particle size and ζ-potential were measured using a Zetasizer Nano ZS instrument (Malvern Instruments Inc., Malvern, UK).

### 3.3. Sonochemical Coating of Cork with AgLNP

Cork granules were sonochemically functionalized with the AgLNP in the presence and absence of the oxidative enzyme laccase (Figure 1). Prior to the coating with AgLNP, the cork granules were cleaned by a sequential soaking process using HCl (pH 2.0), distilled water, NaOH (pH 10.0), and 96% EtOH, followed by three rinses with distilled water. Finally, the granules were dried at 60 °C for 12 h. For the sonochemical coating of the cork particles, a Ti-horn (20 kHz) ultrasonic transducer was introduced into a reaction vessel containing 7 mL of 1 mg/mL of AgLNP and 62.5 mL of 0.1 M succinic acid/succinate buffer (pH 5.0) in the absence (Control) or presence of laccase (final concentration of 0.5 U/mL for each g of cork granules). The coating was performed at 50 °C for 30 min (Figure 1).

### 3.4. Physicochemical and Antimicrobial Characterization of the AgLNP−Cork Nanocomposite

Fourier transform infrared spectroscopy with attenuated total reflectance (FTIR-ATR) was used to analyze the surface of the AgLNP-coated cork granules. The FTIR-ATR spectra were collected in the range of 4000–600 cm^−1^ using a Perkin-Elmer Spectrum 100 (Waltham, USA) equipped with a universal ATR sampling accessory. A total of 34 scans per sample were performed. The antimicrobial performance of AgLNP functionalized cork particles in liquid media was evaluated against *E. coli* ATCC 25922 and *S. aureus* ATCC 25923. For this purpose, 0.80 ± 0.05 g of AgLNP-coated cork were added with 5.0 mL of a bacterial suspension (OD_600_ = 0.01) and incubated at 37 °C under agitation (230 rpm) for 24 h, 72 h, and 120 h. At the end of each incubation time, the number of viable bacteria was determined by plate-counting on selective agar media (i.e., Baird-Parker agar for *S. aureus* and TBX for *E. coli*). The plates were incubated at 37 °C for 24 h in a culture chamber to allow colony growth. The results were expressed as log_10_ of colony forming units (CFU) per milliliter of sample (Log CFU/mL).

### 3.5. Determination of AgLNP-Cork Nanocomposite Disinfection Efficiency in a Water Filtration System

**Figure 7:**
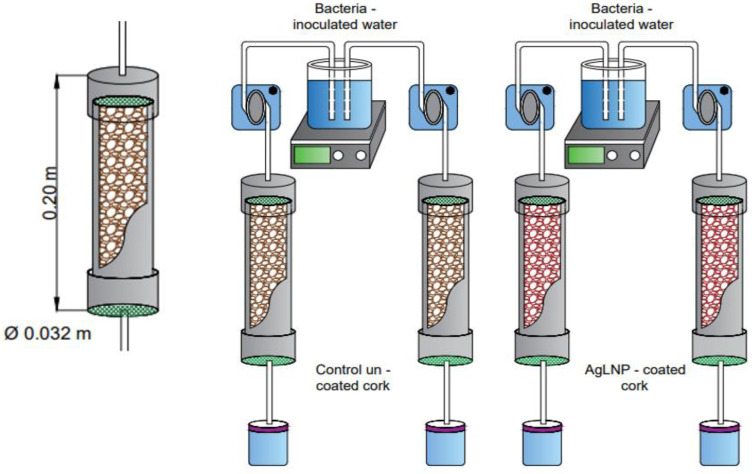
Experimental setup consisting of two filtering cartridges packed with uncoated cork (control) or AgLNP-cork nanocomposite material.

A water filter cartridge system (50 mL total useful capacity) was built as previously described by [40]. Briefly, PVC pipes (diameter 3.2 cm; length 20 cm) were filled with non-coated cork (control) or AgLNP-coated cork and closed up with a polyethylene grid at the top and a geotextile piece at the bottom (Figure 7). A peristaltic pump (Etatron DS, Italy) was used to drive a bacterial suspension from an inlet tank (under constant stirring at 250 rpm to avoid cells sedimentation) to a collection reservoir (Figure 7). The disinfection efficiency of the AgLNP-cork nanocomposite material was evaluated against *E. coli* and *B. cereus*. Separate experiments were performed for each test microorganism. Water samples contaminated with *E. coli* (ca. 10^7^ CFU/mL) or *B. cereus* (ca. 10^7^ spores/mL) were pumped from the inlet tank at a constant flow rate of 3.33 mL/min. Once the filter filled, the flow was stopped, and microorganisms were left in contact with the filtering media at different residence times of 1, 4, 6, 16, 24, and 48 h. At the end of each assay, the flow was restarted and the total useful volume of the filter (50 mL) was collected and sampled to determine the number of bacteria. To estimate the concentration of viable *E. coli* both inlet and outlet water samples were serially diluted in saline solution. Then, aliquots of 10–100 µL were plated on TBX agar and incubated at 37 °C for 24 h for colony counting. On the other hand, the number of *B. cereus* spores per mL of sample was estimated using a Neubauer chamber. Viable spores were counted by plating in nutritive TSA and incubated at 37 °C for 24 h. Finally, the antimicrobial efficiency at each residence time was expressed as percentage (%) of reduction as follows:

$$Reduction\;\left(\%\right)=\frac{\left(A-B\right)}{A}*100$$

where *A* represents the initial concentration (inlet) of the test microorganism and *B* represents the concentration of surviving bacteria after treatment (effluent) both expressed as CFU/mL. Cartridges filled with uncoated cork were used as control. All the experiments were performed in duplicate (two filter cartridges per water recirculating system).

### 3.6. Scanning Electron Microscopy

The presence of adhered bacteria in both AgLNP-coated cork and uncoated cork was examined by scanning electron microscopy (SEM). At the end of the sixth disinfection cycle, bacteria potentially adhered to the cork filter media were fixed by immersion in a 4% formaldehyde solution prepared in PBS (pH 7.0) for 30 min, in order to preserve cells’ structure. Then, samples were twice washed with PBS to remove excess formaldehyde, and sequentially soaked for 10 min in 25, 50, 75 and 96% EtOH, respectively. After the dehydration procedure, the cork samples were cryo-fractured by immersion in liquid nitrogen, mounted on bronze stubs, and coated with a gold layer (15 min, 70 mTorr) before obtaining the SEM images. An AMR-1000 scanning electron microscope (Leitz, Germany) was used to analyze the surface of the modified and unmodified cork. All samples were examined using an accelerating voltage of 50 kV and magnifications of 3000× and 10,000×.

### 3.7. Statistical Analysis

Analysis of variance (ANOVA) was performed using the SigmaStat 3.5 program (Systat Software Inc., USA) to compare the data obtained from the different antimicrobial analyses. Student’s *t*-test and Tukey’s honestly significant difference *post-hoc* test at a significance level of 95% (*p* < 0.05) was used to determine significant differences.

## 4. Conclusions

In this study, an antimicrobial nanocomposite material was obtained by a simultaneous sonochemical and laccase catalyzed coating of AgLNP onto cork particles. The antibacterial studies showed that AgLNP-coated cork efficiently inhibited the growth of *E. coli* and *S. aureus* in liquid medium. In addition, the antimicrobial cork proved to be highly efficient for water disinfection acting as a filter cartridge in a point-of-use device. A complete elimination of *E. coli* was achieved within a residence time of 6 h onwards. In addition, a maximal 2.5-log reduction of resistant *B. cereus* spores was achieved after 16–48 h water treatment with the AgLNP-coated cork as the filter medium. Therefore, the presented results prove that ultrasound coating using laccase is a fast, reliable, and sustainable method of AgLNP immobilization onto cork surface, envisioning the potential use of cork by-products in field-scale biofilter application for water disinfection.

## Data Availability

All data generated or analysed during this study are included in this manuscript and its Supplementary Materials.

## Supplementary Materials

The following supporting information can be downloaded at: https://www.mdpi.com/article/10.3390/ijms231911679/s1.
