# Supplementary material for: Sono-Enzymatically Embedded Antibacterial Silver-Lignin Nanoparticles on Cork Filter Material for Water Disinfection
Source: Int J Mol Sci. 2022 Oct 2;23(19):11679. doi: 10.3390/ijms231911679 (PMC9569633; doi:10.3390/ijms231911679)
Supplement: Supplementary file 1 [file ijms-23-11679-s001.zip › ijms-1918252-supplementary.pdf]

International Journal of *Molecular Sciences*

MDPI

Supplementary Materials

Sono-Enzymatically Embedded Antibacterial Silver-Lignin Nanoparticles on Cork Filter Material for Water Disinfection

Lizeth Bermeo [1,2], Kristina Ivanova [3], Leonardo Martín Pérez [1,4,5], Eva Forés [1], Sílvia Pérez-Rafael [3], Juan C. Casas-Zapata [2], Jordi Morató [1] and Tzanko Tzanov [3,*]

[1] UNESCO Chair on Sustainability, ESEIAAT, Universitat Politècnica de Catalonian, 08222 Terrassa, Spain
[2] Grupo de Investigación Ciencia e Ingeniería en Sistemas Ambientales (GCISA), Facultad de Ingeniería Civil, Departamento de Ing. Ambiental, Universidad Del Cauca, Calle 5 No. 4-70, Popayán 190002, Colombia
[3] Grup de Biotecnologia Molecular i Industrial, Departament d'Enginyeria Química, Universitat Politècnica de Catalunya, 08222 Terrassa, Spain
[4] Instituto de Investigaciones en Ingeniería Ambiental, Química y Biotecnología Aplicada (INGEBIO), Facultad de Química e Ingeniería del Rosario, Pontificia Universidad Católica Argentina (UCA), Av. Pellegrini 3314, S2002QEO Rosario, Santa Fe S2002lrk, Argentina
[5] Consejo Nacional de Investigaciones Científicas y Técnicas (CONICET) and Facultad de Ciencias Bioquímicas y Farmacéuticas, Universidad Nacional de Rosario (UNR), Suipacha 531, S2002LRK Rosario, Santa Fe S2002lrk, Argentina
* Correspondence: tzanko.tzanov@upc.edu

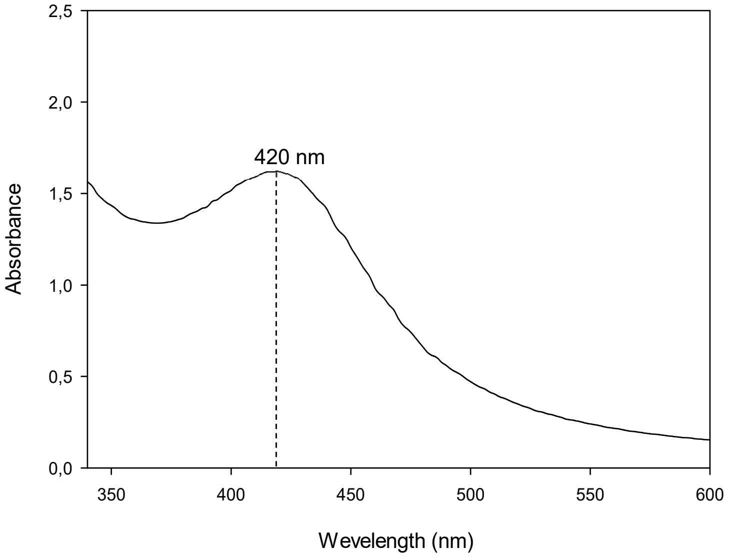

Figure S1. UV−vis spectrum absorption spectrum of the hybrid Ag-lignin nanoparticles (AgLNP) synthesized at the present study.

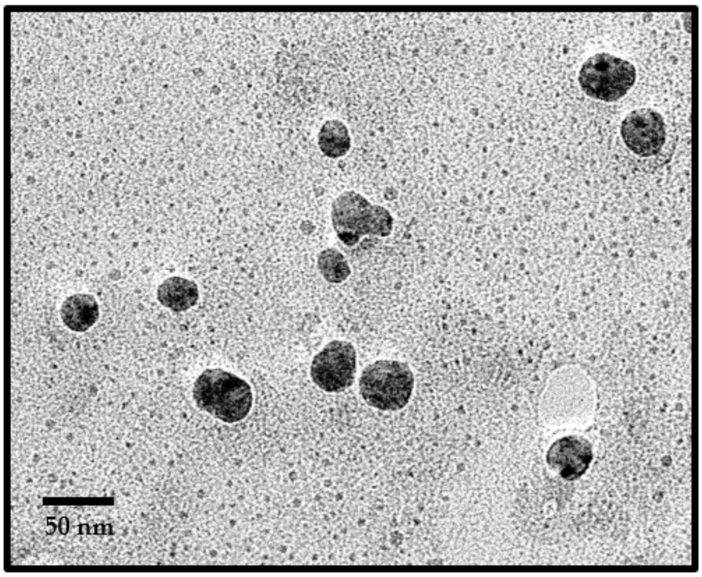

Figure S2. Representative TEM images showing the size and morphology of the AgLNP. Note the regular spherical shape of the nanoparticles with rather similar diameters of the AgLNP.
